# Efficacy and mechanisms of lumbopelvic manipulation combined with Baduanjin exercise for postpartum low back pain: a study protocol for a randomized controlled trial

**DOI:** 10.3389/fmed.2026.1889797

**Published:** 2026-08-31

**Authors:** Guofang Huang, Yuxian Xiong, Xikui Zhang, Yanjun Hou, Jiamin Chen

**Affiliations:** 1 Fujian University of Traditional Chinese Medicine Affiliated Rehabilitation Hospital, Fuzhou, Fujian, China; 2 Fujian Provincial Key Laboratory of Rehabilitation Technology, Fuzhou, Fujian, China; 3 Longyan Ruixin Clinic, Longyan, Fujian, China; 4 Department of Outpatient Services, Fujian Second People's Hospital, Fuzhou, Fujian, China

**Keywords:** Baduanjin, lumbopelvic manipulation, postpartum low back pain, randomized controlled trial, study protocol, surface electromyography

## Abstract

**Introduction:**

Postpartum low back pain (PLBP) is common among puerperal women, with complex etiology involving spinal-pelvic changes and reduced core stability. It often becomes chronic, impairing quality of life and mental health. Non-pharmacological therapies are widely used and show good outcomes, but high-quality RCTs are limited, and objective evidence of core muscle activation after combined manual therapy and Baduanjin is lacking. Baduanjin is a traditional Chinese mind-body exercise that uses gentle movements and coordinated breathing to enhance core stability, making it particularly suitable for postpartum women. Previous studies have demonstrated its effectiveness in reducing pain and improving function in patients with chronic low back pain, with preliminary evidence also supporting its benefits in the postpartum population. This gap undermines the protocol's credibility and clinical uptake. Therefore, this study aims to evaluate the efficacy of lumbopelvic manipulation plus Baduanjin for PLBP, and to explore neuromuscular mechanisms using surface electromyography (sEMG).

**Methods and analysis:**

This single-center, assessor-blinded RCT will enroll 50 participants randomized 1:1 to control (health education + lumbopelvic manipulation) or experimental group (same plus Baduanjin). The control group will receive lumbopelvic manipulation (15 min/session, three times per week), while the experimental group will receive the same manipulation plus supervised Baduanjin exercise (12 min/session, three times per week) for 4 weeks. The 4-week intervention is followed by follow-up assessments at weeks 8 and 12. Primary outcome: pain intensity (Numerical Pain Rating Scale, NPRS). Secondary outcomes: functional disability (Oswestry Disability Index, ODI), quality of life (SF-36), and sEMG parameters (%MVIC and mean power frequency, MPF) of the lumbar extensors (erector spinae and multifidus) and abdominal wall muscles (rectus abdominis, external/internal oblique, and transversus abdominis). Assessments at baseline (week 0) and week 4; NPRS, ODI, SF-36 also at weeks 8 and 12.

**Discussion:**

This trial will evaluate the efficacy of lumbopelvic manipulation combined with Baduanjin exercise for postpartum low back pain, a population with limited medication options during lactation. The inclusion of sEMG alongside patient-reported outcomes will provide objective evidence on neuromuscular changes, extending current evidence that has relied primarily on subjective measures.

**Clinical Trial Registration:** https://itmctr.ccebtcm.org.cn, identifier: ITMCTR2025001056.

## 1 Introduction

Postpartum low back pain (PLBP) is defined as recurrent or continuous pain localized to the lumbar and lumbosacral spine, with or without referral to the gluteal region, that occurs after childbirth (1). This condition significantly impairs a mother's ability to perform daily activities and care for her infant, serving as a major contributor to postpartum disability (2). Miladi et al. (1) reported that approximately 53% of women suffer from this pain during the early postpartum period, whereas Alqabbani et al. (2) demonstrated that the prevalence can reach up to 71% within the first year after delivery. Moreover, Bergström et al. (3) found that a considerable proportion of women still experience persistent or recurrent pain up to 14 months postpartum. Although previous studies have identified risk factors such as a history of low back pain, effective evidence-based non-pharmacological interventions for PLBP remain scarce (1), and the underlying mechanisms are not yet fully understood.

Interventions for spinal and pelvic-derived low back pain are generally classified into three categories: pharmacological, non-pharmacological, and invasive procedures (4). Despite this classification, PLBP as a distinct subgroup remains inadequately managed (5). As most patients are breastfeeding, medications may cause adverse effects in infants via breast milk, and many drugs are contraindicated during lactation (6). Therefore, non-pharmacological therapies are considered the first-line treatment for PLBP (7). Although PLBP shares some clinical features with pelvic girdle pain (PGP), the two conditions are distinct in terms of pain location, aggravating factors, and underlying biomechanical mechanisms. Focusing on PLBP as a specific clinical entity allows for a more homogeneous study population and clearer interpretation of the intervention effects on lumbar core muscle function.

Manual therapy can rapidly correct joint malalignment, relieve pain, and improve range of motion, making it a common clinical approach. Nevertheless, manual therapy alone often provides only short-term relief and has limited effects on improving active stability in patients with chronic non-specific low back pain (8). Similar findings have been reported in postpartum populations, although the evidence remains limited (9). In contrast, exercise therapy focuses on neuromuscular control and core stability reconstruction (10). Through active muscle contraction training, it enhances the strength of lumbopelvic stabilizers (e.g., multifidus and erector spinae), thereby improving the dynamic stability of the lumbopelvic region (11). Various forms of exercise therapy—including walking, yoga, Pilates, strengthening exercises, and core stabilization training—have been investigated for managing low back pain, with systematic reviews supporting their beneficial effects on pain reduction and functional improvement (9). However, many of these approaches may be challenging for postpartum women due to the need for specialized equipment, high physical demands, or restrictions during the early recovery period (12). In contrast, Baduanjin is a traditional Chinese mind-body exercise that requires no equipment and consists of gentle, low-impact movements coordinated with breathing. Its focus on core stability and neuromuscular control, combined with its ease of learning and home-based practice, makes it particularly accessible for postpartum women. In our previous study, Baduanjin significantly reduced pain and improved proprioception in patients with postpartum low back pain (13), supporting its potential in this population.

Despite these findings, existing studies on PLBP interventions have notable limitations. Most investigations report only subjective outcomes (e.g., pain scores and functional questionnaires) and lack objective assessments of muscle activation levels (14). Moreover, although both manual therapy and exercise have demonstrated benefits independently, emerging evidence suggests that combining these approaches may yield additional therapeutic gains. Our previous study, for example, found that Baduanjin combined with routine lumbopelvic manipulation significantly improved pain and proprioceptive function compared with manipulation alone in patients with postpartum low back pain (13). Conceptually, manual therapy is thought to correct joint malalignment and alleviate pain, thereby creating a more favorable biomechanical environment, whereas Baduanjin exercise targets neuromuscular control and core stability. Their combination may thus offer complementary benefits through a “prepare-then-train” sequence (15). However, the specific synergistic mechanisms of combined manual therapy and Baduanjin exercise remain incompletely understood, and studies systematically evaluating such combinations are still limited (16).

Given this background, the present study integrates lumbopelvic manipulation with Baduanjin exercise. In addition to subjective measures (Numerical Pain Rating Scale, NPRS; Oswestry Disability Index, ODI), surface electromyography (sEMG) will be used to objectively quantify changes in muscle activation and fatigue resistance of the lumbopelvic stabilizers—including the erector spinae, multifidus, rectus abdominis, external oblique, internal oblique, and transversus abdominis—using parameters including %MVIC (normalized root mean square expressed as a percentage of maximum voluntary isometric contraction) and mean power frequency (MPF). A comprehensive literature search of PubMed, Web of Science, and CNKI databases using the keywords “lumbopelvic manipulation,” “Baduanjin,” “postpartum low back pain,” and “surface electromyography” revealed no previous studies specifically investigating this combination. To the best of our knowledge, limited evidence exists regarding the combination of lumbopelvic manipulation with Baduanjin exercise for PLBP using sEMG as an objective outcome measure. Specifically, it remains unclear whether combining lumbopelvic manipulation with Baduanjin exercise can improve core muscle activation and neuromuscular function in postpartum women, as most existing studies have relied on subjective outcomes and have not systematically evaluated this combined approach.

## 2 Methods

### 2.1 Trial design

This is a single-center, assessor-blinded, randomized controlled trial in which 50 eligible participants will be randomly assigned in a 1:1 ratio to receive either the control or the experimental intervention. The study will last 12 weeks in total, consisting of a 4-week therapeutic intervention phase (weeks 0–4) followed by an 8-week follow-up period (weeks 4–12).

**Figure 1:**
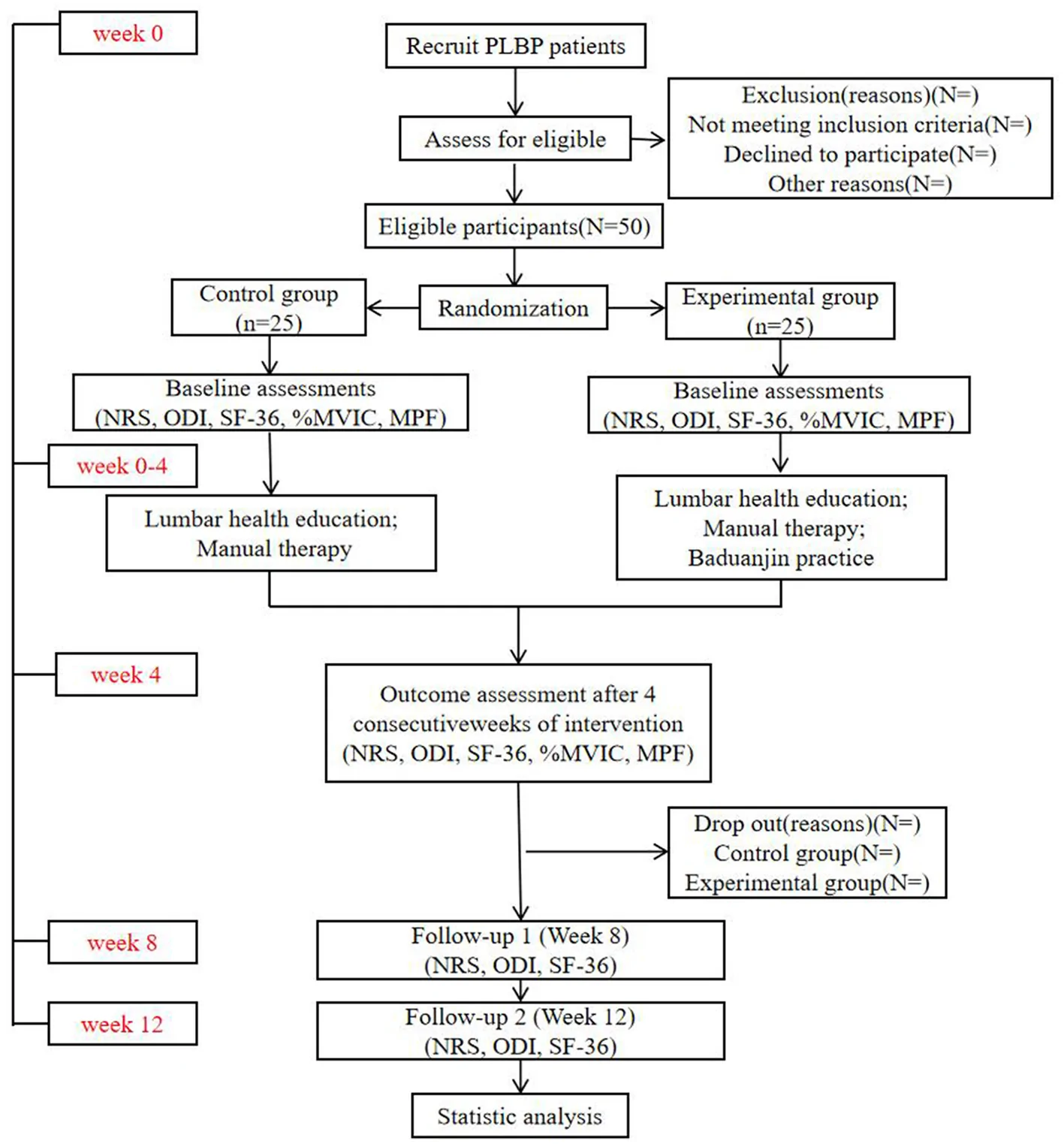
Trial flow diagram.

This trial was registered with the International Traditional Medicine Clinical Trial Registry (ITMCTR2025001056) on 22 April 2025. The protocol conforms to the SPIRIT 2013 reporting guidelines, with the completed checklist provided in the Supplementary material. Figure 1 presents the trial flow diagram, and Table 1 outlines the comprehensive study schedule.

**Table 1:** Trial schedule.

Study period	Enrolment	Intervention	Follow-up	
Time point	Baseline (Week 0)	Week 4	Week 8	Week 12
Eligibility screen	√			
Informed consent	√			
Randomization/allocation	√			
Intervention manipulation		1–4 weeks		
NPRS	√	√	√	√
ODI	√	√	√	√
SF-36	√	√	√	√
sEMG	√	√		
Adverse events monitoring	Any time during the intervention and follow-up			

√, required; NPRS, Numerical Rating Scale; ODI, Oswestry Disability Index; SF-36, 36-Item Short Form Health Survey; sEMG, Surface electromyography.

The primary objective of this study is to evaluate the efficacy of lumbopelvic manipulation combined with Baduanjin exercise for reducing pain and improving functional outcomes in patients with postpartum low back pain. The secondary objective is to explore the neuromuscular mechanisms underlying this combined intervention using surface electromyography.

### 2.2 Ethics approval and consent to participate

This study was approved by the Ethics Committee of the Rehabilitation Hospital Affiliated to Fujian University of Traditional Chinese Medicine (approval No. 2025KY-005-01, dated 31 March 2025). The research will be conducted in accordance with the Declaration of Helsinki (2013 revision) and the relevant regulations of the National Health Commission of the People's Republic of China. All eligible participants will receive a full explanation of the study's purpose, procedures, potential risks and benefits, and their right to withdraw at any time without penalty. Written informed consent will be obtained from each participant prior to any study-specific procedures. The informed consent form will be signed and dated by both the participant and the investigator, with one copy retained by the participant. All participant data will be anonymized and stored in a password-protected electronic database accessible only to the research team. These procedures comply with the relevant data protection regulations of the People's Republic of China, including the Personal Information Protection Law.

### 2.3 Recruitment

This study will be conducted at the Department of Pelvic Floor Rehabilitation and the Department of Tuina of the Rehabilitation Hospital Affiliated to Fujian University of Traditional Chinese Medicine. A multimodal recruitment strategy, incorporating social media platforms (WeChat) and posters displayed in hospitals and community service centers, will be employed to ensure sufficient participant enrollment. Recruitment is expected to take approximately 4–6 months, with the goal of enrolling 50 eligible participants.

### 2.4 Eligibility criteria

#### 2.4.1 Diagnostic criteria for postpartum low back pain

Given the absence of standardized diagnostic criteria specifically for PLBP, we adapted the diagnostic framework from contemporary clinical practice guidelines for the management of low back pain (17). All of the following conditions must be met to ensure the specificity of the diagnosis within the postpartum population:

(1) Onset of low back pain within one year after delivery (18);
(2) Pain localized below the 12th rib and involving the lumbar and lumbosacral spine, with or without referral to the gluteal region;
(3) Pain predominantly dull or aching in nature;
(4) Aggravation by prolonged sitting or standing, heavy lifting, or bending, and relief by rest or treatment;
(5) Imaging examinations (e.g., X-ray or MRI) may be performed when clinically indicated to rule out serious underlying pathologies (e.g., fracture, infection, or malignancy).

Participants with predominant features of pelvic girdle pain—such as pain localized to the sacroiliac joints or pubic symphysis, aggravated by weight-bearing activities—will be excluded to ensure a homogeneous PLBP population.

#### 2.4.2 Inclusion criteria

(1) Participants will be aged 20 to 40 years (inclusive) (19); (2) Participants will have an NPRS score of ≥ 3 at baseline, indicating at least mild to moderate pain intensity, to ensure sufficient room for improvement and clinical relevance. (3) Participants will be primiparous or secundiparous women who have experienced low back pain within 1 year after delivery; (4) Participants will voluntarily enroll in the study and sign the informed consent form; (5) Participants will not use any unrelated physiotherapy or related medications during the treatment period.

The age range of 20–40 years was chosen to focus on the typical reproductive age window and to minimize confounding from age-related physiological differences that could influence treatment outcomes. Similarly, the inclusion was restricted to primiparous and secundiparous women to reduce heterogeneity arising from cumulative biomechanical and neuromuscular changes associated with higher parity, which may affect lumbopelvic stability and treatment responses. While we acknowledge that these criteria may limit generalizability, they were implemented to enhance the internal validity of this proof-of-concept study.

#### 2.4.3 Exclusion criteria

(1) Lumbar tuberculosis, lumbar spinal stenosis, or a history of lumbar spine surgery;
(2) Presence of severe systemic comorbidities, including uncontrolled hypertension, diabetes mellitus with end-organ complications, severe cardiovascular or cerebrovascular disease, or other conditions that, in the investigator's judgment, would contraindicate participation or interfere with the study procedures;
(3) Special populations, including individuals with major psychiatric disorders (e.g., schizophrenia, bipolar disorder, or severe major depression) that, in the investigator's judgment, would impair their ability to provide informed consent, comply with the study protocol, or complete the outcome assessments; deafness, or mutism;
(4) Current participation in other clinical trials.

#### 2.4.4 Withdrawal/dropout criteria

(1) Participants are found not to meet the inclusion criteria after enrollment; (2) Adherence to the intervention protocol falls below 80% (i.e., missing more than 2 of the 12 scheduled manipulation sessions, or more than 5 of the 28 scheduled Baduanjin sessions), without a valid reason documented by the investigator. Missed sessions may be rescheduled at the discretion of the investigator when the absence is due to unavoidable circumstances (e.g., acute illness or family emergency), but such rescheduling does not reset the adherence count; (3) Participants show poor compliance, including changes to or receipt of other relevant treatments during the trial; (4) Participants request to withdraw or voluntarily withdraw from the trial during the study period.

### 2.5 Randomization

Eligible participants will be randomly assigned in a 1:1 ratio to either the control or experimental group using a computer-generated block randomization sequence with a block size of 4 or 6 (randomly varying). The randomization sequence will be generated by a designated biostatistician independent of the study team using SPSS.

To ensure allocation concealment, a research coordinator not involved in participant recruitment will prepare sequentially numbered, opaque, sealed envelopes. Each envelope will contain the group assignment corresponding to the participant's sequential number. Envelopes will be opened in sequential order only after the participant has provided written informed consent, completed all baseline assessments (including demographic characteristics, pain intensity measured by the Numerical Rating Scale [NPRS], functional status assessed by the Oswestry Disability Index [ODI], and quality of life evaluated by the 36-Item Short Form Health Survey [SF-36]), and met all eligibility criteria. The group assignment will be recorded immediately thereafter.

### 2.6 Blinding

Given the nature of manual therapy and Baduanjin exercise, blinding of participants and treating therapists will not be feasible. However, outcome assessors, data collectors, and the statistician will remain blinded to group allocation throughout the trial. Unblinding will be permitted only in the event of a serious adverse reaction. Should unblinding occur, the following procedures will be followed: (1) the investigator who initiated the unblinding will immediately document the date, time, reason, and the identity of the person who was unblinded; (2) the unblinding event will be recorded in the participant's case report form and in a separate Unblinding Log maintained by the principal investigator; and (3) the principal investigator will notify the institutional ethics committee of the unblinding event within 24 h. The participant may continue in the trial at the discretion of the investigator and the ethics committee.

### 2.7 Interventions

#### 2.7.1 Control group intervention

Participants in the control group will receive (1) health education on low back pain management, which will cover posture guidance, weight control, and proper techniques for daily activities such as breastfeeding, lifting, sitting, and standing, and (2) lumbopelvic manipulation therapy.

The manipulation therapy will be performed by a licensed therapist with at least 5 years of experience in musculoskeletal and postpartum rehabilitation. Before each session, the therapist will palpate key bony landmarks—including the anterior superior iliac spines (ASIS), iliac crests, posterior superior iliac spines (PSIS), ischial tuberosities, pubic symphysis, and lumbar spinous and transverse processes—to identify pelvic asymmetry and lumbar spine malalignment.

The treating therapist will perform the manipulation, while a separate assessor (also a licensed therapist, blinded to the treatment session number) will perform the reassessment of bony landmarks after each step to determine whether symmetry has been achieved.

**Figure 2:**
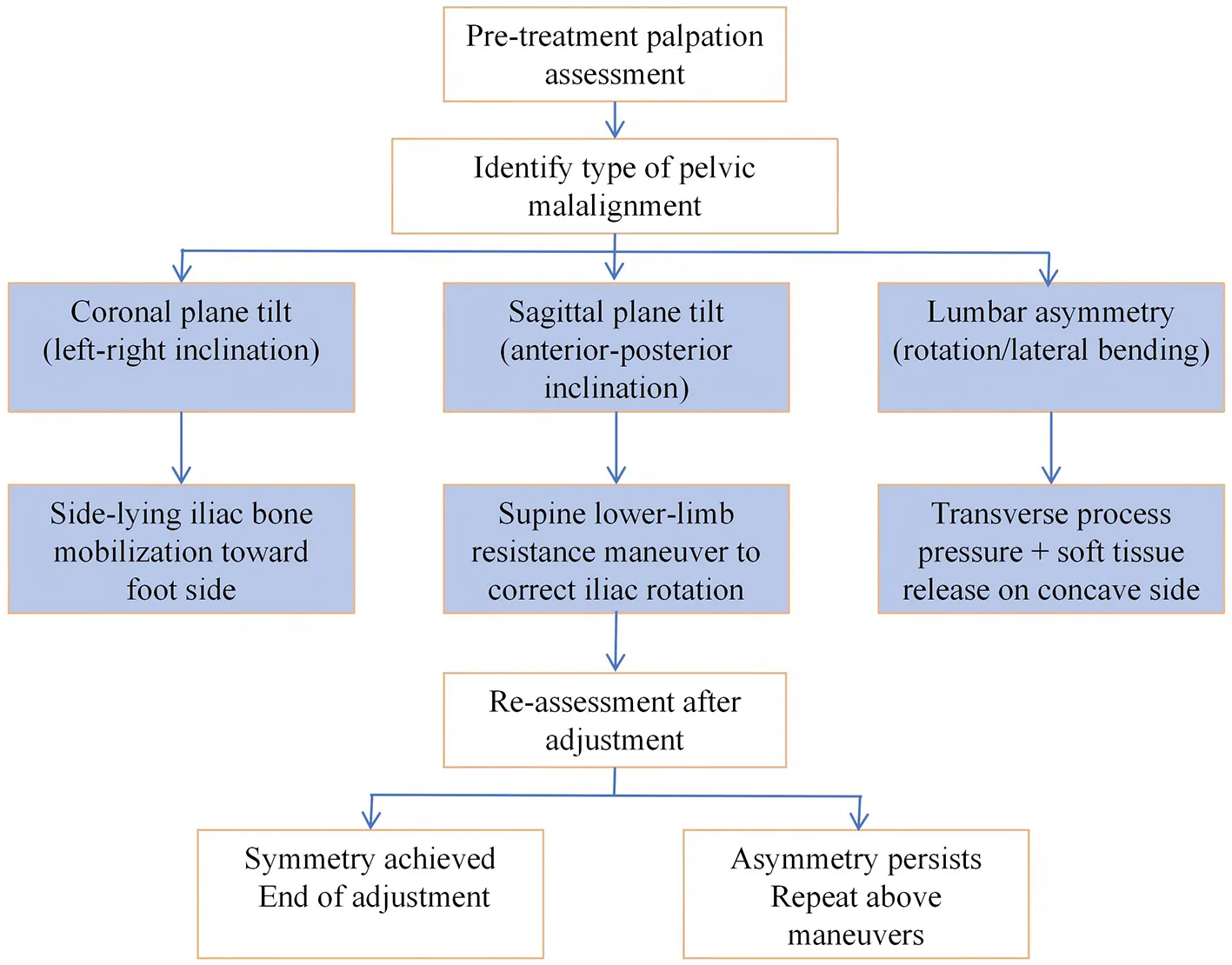
Flowchart of the manual therapy.

The theoretical rationale for these manipulative techniques—including the biomechanical and neurophysiological basis of pelvic micro-adjustment and segmental lumbar mobilization, their mechanisms of action, and their adaptation for postpartum women—is provided in Supplementary file 1, along with full reference citations. The detailed step-by-step manipulation protocol, including specific patient positioning, therapist hand placement, force application, and adjustment criteria for each component, is also provided in Supplementary file 1 to enhance reproducibility while keeping the main text concise. Figure 2 presents the flowchart of this manual therapy procedure.

The treatment will be administered three times per week in 15-min sessions over four consecutive weeks. To ensure consistency, all treating therapists will undergo a standardized training program, and technique performance will be monitored through regular case reviews and video-based assessments.

#### 2.7.2 Experimental group intervention

Participants in the experimental group will receive the same intervention as the control group (health education plus lumbopelvic manipulation), combined with Baduanjin exercise. The Baduanjin protocol will follow the standard version released by the General Administration of Sport of China in 2003 (20). All sessions will be conducted in a quiet, well-ventilated room under the supervision of a trained rehabilitation therapist with a valid rehabilitation therapist license and at least 5 years of clinical experience in postpartum rehabilitation. The full set of eight movements will be performed twice, with a 3-min rest between sets; the eight movements and their key points for postpartum women are summarized in Table 2 each supervised session will last 12 min and will be completed once daily on the days when participants attend the treatment sessions (i.e., three times per week). The frequency of three supervised sessions per week was chosen for two main reasons. First, it aligns the Baduanjin practice with the lumbopelvic manipulation sessions, ensuring standardized delivery of the combined intervention. Second, requiring daily on-site attendance would impose a substantial burden on postpartum women who are caring for newborns, potentially compromising adherence.

**Table 2:** The eight movements of the optimized Baduanjin protocol and key points for postpartum women.

Movement	Name	Key point for postpartum women
1	Holding the hands high with palms up	Avoid excessive lumbar extension; engage core muscles
2	Drawing the bow to shoot the vulture	Keep shoulders relaxed; maintain a neutral pelvic position
3	Holding one arm aloft	Control breathing; avoid trunk twisting
4	Looking backwards	Rotate from the thoracic spine, not from the lumbar spine
5	Swinging the head and tail	Coordinate hip movement; avoid deep squatting
6	Holding the feet to strengthen the kidneys	Keep slight knee flexion; avoid rounding the lower back
7	Clenching fists and glaring angrily	Keep the pelvic floor relaxed; avoid breath-holding
8	Bouncing on the toes	Land softly; maintain core tension throughout

To monitor correct exercise performance, the therapist will provide standardized verbal cues during each session (e.g., “breathe deeply into your lower abdomen and gently draw your navel toward your spine,” “maintain a neutral pelvic position without arching your lower back”). Participants will receive weekly one-on-one assessments of movement quality; signs of inadequate core engagement—such as visible abdominal bulging, excessive lumbar lordosis, or breath-holding during movements—will be documented and addressed through individual instruction. Participants will be instructed not to perform additional Baduanjin practice outside the supervised sessions, and adherence to this restriction will be monitored through daily exercise logs. This restriction was implemented to ensure that the intervention effect can be attributed to the prescribed combined protocol rather than to unsupervised variable-dose practice. By standardizing the frequency, intensity, and supervision of Baduanjin sessions across participants, we minimize confounding from differential home practice, thereby enhancing the internal validity of the trial. During the follow-up period (weeks 4–12), participants will be instructed not to resume Baduanjin practice outside the study protocol. Adherence to this restriction will be monitored through the same daily exercise logs maintained during the intervention phase, which will be reviewed at each follow-up visit (weeks 8 and 12).

### 2.8 Outcome measures

The primary outcome is pain intensity, measured using the Numerical Pain Rating Scale (NPRS). Secondary outcomes include functional disability assessed by the Oswestry Disability Index (ODI), health-related quality of life measured by the 36-Item Short Form Health Survey (SF-36), and neuromuscular function evaluated through surface electromyography (sEMG) parameters: %MVIC (normalized root mean square expressed as a percentage of maximum voluntary isometric contraction) and mean power frequency (MPF). Assessments will be conducted at baseline (week 0) and immediately post-intervention (week 4). The NPRS, ODI, and SF-36 will also be reassessed at weeks 8 and 12.

#### 2.8.1 Numerical pain rating scale (NPRS)

The NPRS is a standardized instrument for assessing pain intensity in patients with postpartum low back pain, utilizing an 11-point numerical rating scale ranging from 0 (no pain) to 10 (worst imaginable pain). Participants select the number that best reflects their current pain level. For chronic low back pain, a 2-point change on the NPRS represents the minimal clinically important difference (MCID) (21).

#### 2.8.2 Oswestry disability index (ODI)

This questionnaire assesses functional disability in patients with postpartum low back pain across ten domains: pain intensity, personal care, lifting, walking, sitting, standing, sleeping, sex life, social life, and traveling. Each domain is scored from 0 to 5, yielding a total raw score between 0 and 50. For analytical purposes, the raw score is multiplied by 2 to produce a standardized percentage score from 0 to 100, with higher scores indicating more severe disability and greater functional limitations in daily activities (22).

#### 2.8.3 36-item short form health survey (SF-36)

The SF-36 comprises 36 items that assess the impact of pain on daily health across eight dimensions and is widely used in clinical research. Scores range from 0 to 100, with lower scores reflecting poorer quality of life (23). In this study, a 30% improvement from baseline in the SF-36 score will be considered clinically meaningful (24).

#### 2.8.4 Surface electromyography (sEMG) signal acquisition and analysis

Surface EMG signals will be recorded from the bilateral erector spinae, multifidus, rectus abdominis, external oblique, internal oblique, and transversus abdominis using a Delsys Trigno wireless sEMG system (2000 Hz sampling rate, 10–500 Hz bandwidth). These muscles were selected as they represent the primary lumbopelvic stabilizers, encompassing both the lumbar extensors and the abdominal wall muscles, whose coordinated activation is essential for core stability. Electrodes will be positioned in accordance with SENIAM guidelines (25). Prior to attachment, the skin will be shaved, abraded, and cleaned.

The testing protocol will comprise two parts: functional tasks and maximal voluntary isometric contraction (MVIC). Measurements will be taken before the intervention and after 4 weeks of training. Functional tasks will include prone trunk extension (erector spinae and multifidus), supine curl-up (rectus abdominis), supine rotational curl-up against resistance (external and internal obliques), and abdominal drawing-in (transversus abdominis). Each movement will be held for 3 s and repeated three times. During MVIC testing, participants will perform resisted movements specific to each muscle (e.g., prone resisted trunk extension and supine resisted curl-up) and sustain the contraction for 3 s, with three repetitions. The order of the functional tasks will be fixed across all participants to ensure consistent testing conditions. A 1-min rest interval will be provided between each functional task to minimize muscle fatigue. MVIC testing will be performed after all functional tasks are completed, with a 2-min rest period before the first MVIC trial to allow for full recovery. For %MVIC calculation, the RMS value from each of the three 3-s functional task repetitions will be averaged, and this mean will be normalized to the highest RMS value recorded during any of the three MVIC trials (i.e., peak MVIC), following standard sEMG normalization practices. For MPF, the mean power frequency will be calculated from the same analysis window (1-s epoch centered on the middle of each 3-s contraction) and averaged across the three repetitions.

Participants will be instructed to perform each task to the best of their ability without exacerbating pain. If a participant reports pain that prevents completion of a task as described, the task may be modified (e.g., reduced range of motion, decreased repetition number, or substitution of a modified curl-up) to allow for data collection while minimizing discomfort. Any modifications will be recorded in the case report form. Participants who are unable to perform a specific task due to pain or physical limitations will not be forced to complete it. This will be documented as a protocol deviation. Data from modified tasks will be excluded from the primary between-group analyses to ensure comparability of the sEMG measurements. These data will be reported descriptively in Supplementary material.

Offline signal processing will involve band-pass filtering (20–450 Hz) and a 50 Hz notch filter. The root mean square (RMS) value will subsequently be calculated. The %MVIC for each functional task will be determined by normalizing the RMS value to the maximum RMS obtained during MVIC. %MVIC will reflect the level of muscle activation. MPF is a sensitive frequency-domain indicator of local muscle fatigue, with a decrease signifying fatigue. In this study, changes in MPF during functional tasks before and after the intervention will be compared to evaluate muscle anti-fatigue capacity.

### 2.9 Safety evaluation and adverse events (AEs)

Participants will be continuously monitored for safety throughout each intervention session. All AEs occurring during the intervention or follow-up period will be recorded on case report forms (CRFs). In the event of an AE, qualified medical personnel will provide immediate symptomatic treatment, regardless of its suspected cause. The CRF will document the AE's onset, manifestations, severity, treatment, and outcome. Safety monitoring will continue until the participant's condition stabilizes or resolves.

A serious adverse event (SAE) is defined as any untoward medical occurrence that results in death, is life-threatening, requires hospitalization or prolongation of existing hospitalization, results in persistent or significant disability/incapacity, constitutes a congenital anomaly/birth defect, or requires medical intervention to prevent permanent impairment or damage. All AEs will be reported to the principal investigator (PI) and the institutional ethics committee for review, which will then determine whether the participant may continue the trial. Study-related SAEs will be compensated in accordance with regulatory and institutional requirements.

Based on clinical experience with these interventions, lumbopelvic manipulation is generally well tolerated, with potential treatment-related effects typically limited to transient muscle soreness or stiffness. To date, no study-related serious adverse events have been observed in our clinical practice. Baduanjin exercise, which consists of gentle, low-impact movements, is considered safe for postpartum women. Any adverse events that do occur will be recorded, managed, and reported according to the procedures described above.

### 2.10 Data management and quality control

All clinical data collected by qualified physicians will be entered into a dedicated electronic database, with participant identities replaced by numerical codes. The principal investigator will be responsible for data quality at the study site. Quarterly investigator meetings will address trial-related issues and ensure procedural consistency. Telephone reminders for scheduled visits and assessments will be provided to participants to improve adherence. To monitor adherence to the protocol restriction on unrelated physiotherapy and medications, the following measures will be implemented: (1) participants will be instructed to maintain a daily log recording any additional treatments or medications used during the intervention period; (2) at each study visit (weeks 4, 8, and 12), participants will be systematically asked about their use of other therapies; and (3) weekly telephone calls will be made to reinforce the protocol requirements. Any deviation from the protocol will be recorded in the case report form and reported to the principal investigator. Participants who fail to comply with the protocol will be considered for withdrawal according to the predefined criteria.

### 2.11 Sample size

The sample size was determined based on the primary outcome, the Numerical Pain Rating Scale (NPRS). The minimal clinically important difference (MCID) of the NPRS among patients with low back pain has been validated as 2 points on the 0–10 scale (21, 24). A between-group difference of 2 points in the change of NPRS from baseline was defined as a clinically meaningful threshold in the present study. We adopted a two-sided significance level of 0.05 and statistical power of 80%, and calculated sample size using the standard formula for the comparison of two independent means. Based on prior studies enrolling comparable low back pain populations (21, 24), the pooled standard deviation (SD) of NPRS change scores was estimated as 2.3. Given a minimal detectable between-group difference of 2 points, this calculation yielded a raw sample size of approximately 22 participants per group. Given the limited sample size of our preliminary pilot data (*n* = 12) leading to uncertain SD estimation, we further conducted a sensitivity analysis to test the robustness of our sample size calculation. When SD varied from 2.0 to 2.5, the unadjusted required sample size per group ranged from 16 to 26 participants prior to dropout adjustment. After accounting for an anticipated 20% participant dropout rate, the adjusted target sample size ranged from 20 to 33 participants per group. Our pre-specified recruitment target of 25 participants per group lies within this valid range, which guarantees adequate statistical power for the primary analysis focused on clinical pain outcomes (NPRS).

### 2.12 Statistical analysis

All statistical analyses will be performed using SPSS version 26.0. Primary analyses will be conducted on an intention-to-treat (ITT) basis, including all randomized participants in their originally assigned groups regardless of protocol adherence or withdrawal. Missing data will be handled via multiple imputation (MI), the gold-standard approach for missing continuous clinical outcome data in randomized controlled trials. Last observation carried forward (LOCF) will not be adopted in the present study, as this single-imputation method is prone to bias and has been widely discouraged in contemporary statistical guidelines. Continuous variables will first be tested for normality using the Shapiro-Wilk test. Normally distributed data will be reported as mean ± standard deviation (SD), whereas non-normally distributed data will be presented as median (first quartile, third quartile) [M (P25, P75)]. For within-group comparisons, paired-sample t-tests will be applied to normally distributed data, and Wilcoxon signed-rank tests to non-normally distributed data. For between-group comparisons at baseline, independent-sample t-tests will be used for normally distributed data, and Mann-Whitney U tests for non-normally distributed data. To compare post-intervention outcomes between the two groups, analysis of covariance (ANCOVA) will be performed, with baseline values as covariates to adjust for potential baseline imbalance. In addition, potential confounding variables—including postpartum duration (months since delivery), age, and baseline outcome values—will be recorded and considered as covariates in the primary analyses where appropriate. Exploratory correlation analyses will employ Pearson or Spearman correlation coefficients, as appropriate, to examine associations among post-intervention pain intensity (NPRS), the Oswestry Disability Index (ODI), the SF-36, core muscle activation (%MVIC), and mean power frequency (MPF). These analyses are hypothesis-generating and will be interpreted with caution; formal corrections for multiple comparisons will not be implemented. All tests will be two-sided, and a *P*-value < 0.05 will indicate statistical significance. Given the exploratory nature of these analyses and the multiple comparisons involved, the results should be interpreted as hypothesis-generating, and any statistically significant findings will require confirmation in future studies.

## 3 Discussion

This study protocol presents a randomized controlled trial evaluating the efficacy and neuromuscular mechanisms of lumbopelvic manipulation combined with Baduanjin exercise for postpartum low back pain. The ‘prepare-then-train' sequence is grounded in clinical experience and theoretical reasoning, although direct comparative evidence is currently limited (15). This study is designed to provide objective sEMG evidence to test this hypothesis.

Given the limited data on postpartum populations and the scarcity of objective neuromuscular assessments in this field (9), our protocol addresses these gaps by combining validated patient-reported outcomes (NPRS, ODI, SF-36) with quantitative sEMG parameters (%MVIC and MPF). This design allows us to assess whether improvements in pain and function are accompanied by objective changes in core muscle activation and fatigue resistance.

Assessing muscle fatigue and endurance is particularly relevant in the postpartum population (26). Pregnancy and childbirth lead to reduced endurance of the core musculature which compromises spinal and pelvic stability (27). Compounding this physiological decline, postpartum women face substantial physical demands, including frequent bending, lifting, and carrying of the infant, as well as breastfeeding, all of which impose repetitive loads on the lumbopelvic region and may accelerate muscle fatigue (28). Therefore, evaluating changes in MPF—a sensitive frequency-domain indicator of muscle fatigue—provides insight into whether the combined intervention improves the ability of the lumbar extensor muscles to resist fatigue during functional tasks, which may be a key mechanism underlying its therapeutic effects.

The inclusion of abdominal muscle sEMG recordings allows for exploratory analyses of the coordinated activation patterns of the lumbopelvic stabilizers, complementing the primary focus on the multifidus and erector spinae muscles. A recent systematic review identified multiple differences in motor control, including altered lumbar motion patterns and muscle activation, in women with pregnancy-related lumbopelvic pain during dynamic tasks (29). While sEMG is well established for assessing paraspinal muscle activity in low back pain, its combined use of both %MVIC and MPF to evaluate a manual-exercise intervention in postpartum low back pain has not been previously reported. To confirm this, we searched PubMed, Web of Science, and CNKI databases using the keywords “lumbopelvic manipulation,” “Baduanjin,” “postpartum low back pain,” “%MVIC,” and “MPF.” The search yielded no studies specifically investigating this combination of outcome measures. Our previous study demonstrated that the combination of lumbopelvic manipulation and Baduanjin significantly improved pain and proprioceptive function in patients with postpartum low back pain (13), suggesting that exploring the neuromuscular mechanisms underlying this combined intervention using objective sEMG parameters is clinically warranted. Given that pregnancy is known to alter lumbar motion patterns and paraspinal muscle responses (30), exploring the neuromuscular effects of such combined interventions is clinically relevant.

Postpartum low back pain (PLBP) often recurs or persists for months after delivery (2). Our follow-ups at weeks 8 and 12 are designed to assess whether the benefits of the combined intervention are sustained after treatment cessation, which is critical for clinical decision-making. Several randomized controlled trials have investigated manual therapy or exercise alone for PLBP, but to our knowledge, few have systematically combined them in a structured manner (16). Our protocol addresses this gap by incorporating sEMG for objective muscle activation assessment and a standardized Baduanjin protocol based on the official version developed by the State General Administration of Sport of China (20). Furthermore, all interventions will be delivered by licensed therapists with standardized training, and a detailed protocol manual is provided as Supplementary material to enhance reproducibility.

If the hypotheses are supported, this study may offer a safe, non-pharmacological option for breastfeeding women with PLBP. This population often faces greater caution with medication use during lactation. The sEMG data may also provide biomechanical evidence for the concept of “bone alignment and tendon flexibility” (Gu Zheng Jin Rou in Chinese)—a traditional Chinese medicine principle emphasizing the interdependence between structural alignment and neuromuscular function. This could facilitate the acceptance of traditional Chinese exercise therapy in Western rehabilitation settings.

Several limitations should be acknowledged. First, due to the nature of manual therapy and Baduanjin exercise, blinding of participants and treating therapists is not feasible. However, outcome assessors and the statistician will remain blinded throughout the trial to minimize detection bias. Second, the follow-up period is limited to 8 weeks after treatment, which precludes the evaluation of very long-term effects. Additionally, sEMG assessments are performed only at baseline and week 4, whereas clinical outcomes are followed to week 12. This difference reflects the aim of capturing immediate neuromuscular adaptations during the active intervention phase while minimizing participant burden, but it limits our ability to assess whether these adaptations are sustained during the follow-up period. Third, sEMG signals are collected only from selected core muscles (erector spinae, multifidus, rectus abdominis, external/internal oblique, and transversus abdominis) and do not include the pelvic floor muscles, which also play a crucial role in lumbopelvic stability. Fourth, the sample size (25 per group) may limit subgroup analyses, such as comparing primiparous vs. multiparous women or different delivery modes. Additionally, the inclusion of only primiparous and secundiparous women may limit the generalizability of our findings to women with higher parity. The correlation analyses examining associations between pain, functional outcomes, and sEMG parameters are exploratory in nature and intended to generate hypotheses for future studies. As these analyses were not prespecified in the trial registration, the results should be interpreted with caution.

Additionally, the generalizability of this protocol to settings where lumbopelvic manipulation and Baduanjin are not routinely taught may be limited. While our therapists are licensed professionals with specialized training in these techniques, implementation in other countries or healthcare systems may require additional training or adaptation. Future studies should consider evaluating the effectiveness of this protocol in diverse settings and exploring training approaches to enhance its global applicability.

Larger multicenter trials are needed to confirm the robustness of these findings. Subgroup analyses comparing cesarean section with vaginal delivery may help identify which patients derive the greatest benefit. The optimal frequency, duration, and intensity of Baduanjin training remain to be determined. Incorporating advanced techniques such as ultrasound imaging to measure muscle thickness or functional near-infrared spectroscopy (fNIRS) to assess cortical activation patterns could offer deeper insight into the neuromuscular mechanisms underlying this combined intervention.
