# Supplementary material for: Efficacy and mechanisms of lumbopelvic manipulation combined with Baduanjin exercise for postpartum low back pain: a study protocol for a randomized controlled trial
Source: Front Med (Lausanne). 2026 Aug 31;13:1889797. doi: 10.3389/fmed.2026.1889797 (PMC13572278; doi:10.3389/fmed.2026.1889797)
Supplement: Supplementary file 1 [file Table_1.docx]

# **Theoretical Basis and Standardized Protocol for Three-Step Pelvic Micro‑Adjustment Combined with Segmental Lumbar Mobilization**

## 1. Theoretical Basis and Procedure for Three-Step Pelvic Micro‑Adjustment

### 1.1 Theoretical Basis for Pre‑Treatment Assessment

The pelvis is the mechanical hub that links the trunk to the lower extremities. Formed by the bilateral ilia, sacrum, coccyx, and the anterior pubic symphysis, it creates a closed osteoligamentous ring. The posterior sacroiliac joints transmit gravitational forces from the trunk and preserve the mechanical integrity of the lumbosacral-pelvic-lower limb chain. These joints are also among the most vulnerable structures in the human kinetic chain to micro-displacement and mechanical dysfunction [1]. When pelvic dysfunction occurs—whether as iliac rotation or sacroiliac joint dysfunction—it typically presents as vertical or rotational displacement of the ilium. This displacement sets off a cascade of consequences: bony landmark asymmetry, muscular imbalance, restricted joint motion, and secondary clinical problems such as low back pain, pelvic tilt, functional leg length discrepancy, and compensatory spinal scoliosis [2].

In this protocol, we use a multi‑landmark palpation system that targets the anterior superior iliac spines (ASIS), iliac crests, posterior superior iliac spines (PSIS), ischial tuberosities, pubic symphysis, and lumbar spinous and transverse processes to identify three‑dimensional pelvic mechanical imbalance. Pelvic dysfunction is classified into three main categories: (1) coronal plane vertical displacement of the ilium, (2) sagittal plane anterior/posterior rotation of the ilium, and (3) horizontal plane rotational malalignment of the ilium [3]. This system enables accurate differentiation of these malalignment types, thereby guiding the sequential three‑step micro‑adjustment protocol and ensuring a diagnosis‑driven treatment approach.

(1) Sagittal plane anterior/posterior rotation of the ilium: When the PSIS and ischial tuberosity are positioned more cranially on one side, while the ASIS and pubic symphysis lie more caudally on the same side, this indicates anterior rotation of the ilium. The opposite pattern—a more caudal PSIS and ischial tuberosity combined with a more cranial ASIS and pubic symphysis—points to posterior rotation. These findings guide the use of lower‑limb resistance correction techniques [4].

(2) Coronal plane vertical displacement of the ilium: Elevation of the unilateral iliac crest, ASIS, PSIS, ischial tuberosity, and pubic symphysis indicates superior gliding of the ilium with impingement at the superior margin of the sacroiliac joint. Conversely, depression of these landmarks signals inferior gliding. These findings serve as the primary indication for the second step of the manipulation [4].

(3) Horizontal plane rotational malalignment of the ilium: This pattern is characterized by anterior/posterior depth asymmetry of the bilateral ASIS—one side protruding anteriorly while the other appears recessed posteriorly—often accompanied by medial convergence of one ASIS and lateral divergence of the other. Additional findings include deviation of the pubic symphysis with unilateral tenderness and horizontal mobility on palpation. Together, these signs suggest horizontal plane rotational displacement of the ilium [4].

The lumbar spinous and transverse processes serve as superior reference landmarks. They help determine whether pelvic malalignment reflects a primary iliac problem, or is instead a secondary pelvic tilt caused by lumbar scoliosis or compensatory trunk deviation. This distinction helps rule out false pelvic asymmetry and improves assessment accuracy [4].

### 1.2 Therapeutic Mechanism of Pelvic Micro‑Adjustment Manipulation

The three‑step pelvic micro‑adjustment protocol combines muscle energy techniques, mobilization with movement, and low‑amplitude joint gapping. The correction follows a sequential logic: sagittal plane correction comes first, followed by coronal plane alignment, then horizontal plane rotational adjustment. Within each plane, the approach proceeds from soft tissue release to joint alignment restoration, and ultimately to global pelvic balance—a stepwise progression toward overall symmetry. The procedure uses low‑intensity, controlled, patient‑active movements and deliberately avoids high‑velocity thrusts. Through the combined effects of muscle energy relaxation, joint gapping, and capsular release, it progressively reduces sacroiliac joint restriction, relieves soft tissue spasm, and resolves pelvic mechanical locking, eventually restoring three‑dimensional pelvic symmetry and mechanical stability.

Step 1 employs muscle energy techniques to correct sagittal plane anterior/posterior rotation of the ilium, balancing the height differences of the bilateral PSIS, ischial tuberosities, ASIS, and pubic symphysis [3]. Step 2 uses directed joint mobilization to release adhesion at the superior margin of the sacroiliac joint, consolidating the coronal plane correction [4]. Step 3 combines resistance‑based muscle balancing with low‑amplitude joint gapping to correct horizontal plane rotational malalignment and minor pubic symphysis displacement, thereby achieving three‑dimensional pelvic realignment [4]. The entire protocol is safe, reproducible, and particularly suitable for mild‑to‑moderate pelvic rotational dysfunction in postpartum women.

### 1.3 Standardized Manipulation Procedure

Prior to treatment, the therapist systematically palpates the ASIS, iliac crests, PSIS, ischial tuberosities, pubic symphysis, and lumbar spinous and transverse processes. This assessment serves to characterize three‑dimensional pelvic symmetry, identify the side and type of dysfunction, and guide the subsequent manipulation.

#### 1.3.1 Step 1: Supine Lower‑Limb Isometric Contraction for Sagittal Plane Correction

The participant lies supine with hips and knees flexed and feet flat on the bed, maintaining a neutral pelvic baseline. The therapist places one hand on the lower leg of the side with the lower ASIS and the other on the thigh of the side with the higher ASIS. The participant is instructed to inhale naturally and, during exhalation, engage the core muscles and gently press the lower limbs against the therapist's hands at approximately one‑third of maximal effort. This contraction is held for 10 seconds and repeated three times [3].

Immediately after each set, the therapist re‑palpates the bony landmarks to determine whether the anterior/posterior rotation has been corrected. If correction remains incomplete, the resistance procedure is repeated; once symmetry is restored, the therapist moves on to the next step.

#### 1.3.2 Step 2: Side‑Lying Iliac Crest Directed Mobilization for Coronal Plane Correction

The participant lies on the side with the higher iliac crest facing upward. The therapist stands in front of the participant, securely grasps the iliac crest with both hands, and applies brief, low‑amplitude, high‑frequency oscillations in a caudal direction (toward the participant's feet) for 30 seconds. This is repeated three times [4].

After completing the mobilization, the participant returns to the supine position, and the therapist re‑palpates the pelvic bony landmarks to assess whether the coronal plane vertical displacement has been corrected. If correction remains incomplete, the mobilization is repeated; once symmetry is restored, the therapist proceeds to the third step.

#### 1.3.3 Step 3: Hip‑Girdle Resistance with Joint Gapping for Horizontal Plane Correction

The participant returns to the supine position with hips and knees flexed and feet flat on the bed. The therapist stands beside the bed, places both hands on the lateral aspects of the participant‘s knees, and instructs the participant to perform resisted hip abduction at approximately one‑third of maximal effort. This contraction is held for 10 seconds and repeated three times, with the goal of balancing bilateral hip musculature [4].

Following the resistance abduction, the therapist places a clenched fist between the participant’s knees and asks the participant to actively adduct the knees, compressing the fist. This maneuver transmits medial compressive forces through the bilateral ilia, helping to release sacroiliac joint impingement and synovial adhesion. An audible joint click may occasionally occur during this procedure, reflecting the cavitation phenomenon that results from sudden changes in intra‑articular pressure. Such a click typically indicates release of joint restriction and restoration of joint mobility, thereby facilitating micro‑adjustment of horizontal plane rotational malalignment and minor pubic symphysis displacement [4].

After completing the mobilization, the participant returns to the supine position, and the therapist re‑palpates the pelvic bony landmarks to assess whether the horizontal plane rotational malalignment has been corrected. If correction remains incomplete, the mobilization is repeated; once symmetry is restored, the procedure concludes.

This protocol follows a closed‑loop “assessment–correction–reassessment” clinical reasoning process, with independent assessors performing re‑evaluation after each step. The number of repetitions is dynamically adjusted based on the individual‘s response to correction. This approach avoids rigid application of a standardized routine and enhances both clinical precision and reproducibility, making it well suited for clinical dissemination and standardization.

## 2. Segmental Lumbar Mobilization

### 2.1 Theoretical Basis and Adaptation for Postpartum Women

Postpartum women experience a range of biomechanical and physiological changes. Increased lumbar lordosis results from the gravid abdominal load, while sustained tension in the erector spinae and quadratus lumborum develops from prolonged postural adaptation during pregnancy [5]. At the same time, elevated relaxin secretion reduces tension in the lumbar facet joints and thoracolumbar ligaments, leading to decreased lumbar spine stability [5]. In addition, pelvic malalignment generates compensatory forces that transmit cephalad to the lumbosacral junction, producing asymmetrical transverse processes and segmental vertebral rotation at L1–L5—a mechanical chain best described as "pelvic dysfunction‑induced lumbar compensatory malalignment" [6].

This segmental lumbar mobilization protocol draws on the tendinomuscular system theory of traditional Chinese medicine (TCM) and follows the classical concept of “tendon displacement and bone malalignment” from the Inner Canon of Huangdi. It integrates traditional soft tissue techniques—including rolling, kneading, pushing, pressing, and vibrating—with lumbar transverse process‑targeted mobilization, and has been specifically adapted for the unique physiological characteristics of postpartum women [7].

Traditional Chinese manipulation alone emphasizes soft tissue relaxation but does not provide precise segmental correction of lumbar transverse processes or vertebral rotation. Conventional spinal manipulation, by contrast, often relies on high‑amplitude rotational thrusts that carry a risk of excessive joint mobility—particularly in postpartum women with ligamentous laxity. Our protocol addresses this by first using TCM soft tissue techniques to release spasmodic lumbar musculature and eliminate compensatory tension. Brief, low‑amplitude, low‑force vertical mobilization is then applied to the asymmetrical transverse processes, following a caudad‑to‑cephalad segmental sequence (L5 to L1) that is consistent with the biomechanical principle of caudad‑to‑cephalad compensatory transmission [8]. This strategy respects the biomechanical vulnerabilities of postpartum women and offers enhanced safety and specificity for managing postpartum low back pain.

### 2.2 Pre‑Treatment Lumbar Myofascial Assessment

#### 2.2.1 Anatomical Assessment Targets and Pathological Correlates

The quadratus lumborum and erector spinae are the primary dynamic stabilizers of the lumbar spine in the coronal and sagittal planes. They also serve as key connecting structures in the pelvic‑lumbar mechanical transmission chain. After pelvic correction, if three‑dimensional alignment has been restored but unilateral quadratus lumborum or erector spinae remains hypertrophied, stiff, or tender to palpation, this indicates persistent myofascial compensatory spasm resulting from prolonged pelvic tilt. Unilateral hypertonicity creates sustained traction on the corresponding lumbar transverse processes and pelvis, leading to lateral vertebral deviation, segmental rotation, and a predisposition to recurrent pelvic malalignment—forming a vicious cycle of “tendon tension‑induced bone malalignment” [8,9].

The erector spinae runs longitudinally along both sides of the lumbar spinous processes. Unilateral hypertrophy or cord‑like induration in this muscle suggests an anterior‑posterior compensatory stress imbalance, predisposing the spine to anterior or posterior vertebral rotation [8,9]. The quadratus lumborum connects the iliac crest to the lumbar transverse processes and is the primary muscle responsible for lateral flexion and coronal plane stability of the lumbar spine. Unilateral tension or spasm in this muscle directly elevates the ipsilateral lumbar transverse processes and ilium, making it the main soft tissue contributor to lumbar transverse process asymmetry and unilateral coronal plane iliac displacement [8,9].

#### 2.2.2 Bilateral Thumb Pad Sequential Palpation Assessment

Bilateral thumb pad palpation from cephalad to caudad, using pointing and pressing techniques, is a classic soft tissue examination method in Chinese tendinomuscular medicine. It aligns well with modern myofascial pain assessment standards [8]. Simultaneous bilateral comparison palpation allows for direct visual differentiation of muscle thickness and tension asymmetry. Findings such as increased thickness, a cord‑like texture, or deep radiating pain on palpation on the affected side suggest the presence of myofascial trigger points [10].

Postpartum muscle spasm typically results from prolonged compensatory mechanisms. Correcting bony alignment without first releasing spasmodic musculature predisposes the patient to rapid recurrence of malalignment [5]. For this reason, myofascial assessment and release are positioned as a preparatory step before lumbar correction. This step eliminates soft tissue traction forces and creates a tension‑free mechanical environment for subsequent transverse process mobilization and vertebral reduction.

#### 2.2.3 Mechanism of Traditional Chinese Soft Tissue Release Techniques

A combination of rolling, kneading, pushing, pressing, and vibrating techniques is applied to areas of muscle tension or pain for approximately 3 minutes at moderate intensity. This approach is grounded in both traditional Chinese medicine and modern rehabilitation principles. From a TCM perspective, rolling and kneading promote qi and blood circulation along the Bladder meridian of the lower back and help relax adhesions. Pointing and pressing directly target acupoints and trigger points to unblock meridians and relieve pain. Vibrating techniques release fascial tension, achieving what is traditionally described as "relaxation of displaced tendons" [7].

### 2.3 Assessment of Lumbar Transverse Process Asymmetry and Segmental Vertebral Rotation

#### 2.3.1 Biomechanical Basis of Lumbar Transverse Process Asymmetry and Segmental Vertebral Rotation

The lumbar transverse processes serve as attachment points for the quadratus lumborum, multifidus, and thoracolumbar fascia [11], and are commonly used as surface landmarks for detecting segmental lumbar vertebral asymmetry in manual therapy practice [12]. Under normal neutral alignment, bilateral transverse processes at the same segmental level lie at the same height. When segmental vertebral rotation occurs, the transverse process on the rotated side may become displaced, resulting in asymmetry [12].

After pelvic correction, compensatory forces may continue to transmit cephalad through the lumbosacral junction [6]. The L5 segment is often the first to be affected, followed sequentially by L4, L3, L2, and L1. Assessment therefore follows a caudad‑to‑cephalad segmental logic (L5 → L4 → L3 → L2 → L1). If transverse process asymmetry persists despite adequate myofascial release, this may indicate primary segmental vertebral rotation or lumbar facet joint restriction, warranting further investigation and targeted mobilization.

#### 2.3.2 Caudad‑to‑Cephalad Segmental Assessment Logic (L5 → L4 → L3 → L2 → L1)

The lumbosacral joint forms the mechanical transition between the pelvis and the lumbar spine [13]. Compensatory forces arising from pelvic imbalance propagate progressively in a caudad-to-cephalad direction along the spine, as caudal segments exert a sequential influence on those located more cranially [6]. Our protocol therefore adopts a caudad-to-cephalad segmental assessment sequence, correcting lower segments before upper ones to prevent persistent compensation from unreduced lower segments undermining the correction achieved at upper levels. This strategy is consistent with the established biomechanical principle of caudad-to-cephalad compensatory transmission [8].

### 2.4 Theoretical Basis for Brief, Low‑Amplitude Lumbar Transverse Process Mobilization

#### 2.4.1 Anatomical and Biomechanical Basis for Positioning and Force Application

The participant remains in the prone position, with the lumbar musculature already relaxed by the preceding soft tissue techniques. The therapist then applies bilateral thumb‑over‑thumb vertical pressure downward onto the elevated transverse process. The force vector is directed perpendicular to the bony plane of the transverse process, allowing direct transmission to the transverse process and the corresponding vertebral body while minimizing soft tissue energy loss. Compared with rotational or high‑amplitude spinal manipulation, this vertical mobilization offers a potentially safer option for postpartum women with hormone‑mediated ligamentous laxity. Such laxity may reduce the protective stability of the intervertebral articulations, although reported adverse events following spinal manipulation in postpartum populations are rare [14].

#### 2.4.2 Mechanism of Brief, Low‑Amplitude Oscillatory Mobilization

Sustained 30‑second, brief, low‑amplitude, rapid vertical mobilization applied to the elevated transverse process employs graded joint mobilization techniques [15]. This low‑amplitude oscillation gently separates restricted lumbar facet joints and relieves synovial impingement [16], thereby correcting segmental horizontal plane rotation.

At the same time, sustained mechanical stimulation of the mechanoreceptors surrounding the transverse process can induce a reflex reduction in muscle tone through spinal reflex mechanisms [17], helping to maintain transverse process symmetry after correction. Immediate re‑assessment following each step—with dynamic adjustment of the number of repetitions—forms an individualized “assessment–release–mobilization–reassessment” closed‑loop correction system.

### 2.5 Overall Synergistic Mechanism of the Lumbar Mobilization Protocol

This lumbar mobilization protocol provides two synergistic advantages. First, soft tissue release precedes joint mobilization: by releasing spasmodic erector spinae and quadratus lumborum before correcting bony malalignment, this approach aligns with the classical Chinese medicine principle of “tendon release before bone correction” [18]. Second, it follows a defined sequence of mechanical transmission: after pelvic correction, the protocol systematically eliminates the caudad-to-cephalad compensatory mechanical chain, thereby addressing the underlying lumbopelvic biomechanical imbalance [4,19].

## 3. Overall Advantages and Theoretical Innovation of the Combined Protocol

This combined protocol—integrating the three‑step pelvic micro‑adjustment with segmental lumbar mobilization—rests on a dual theoretical framework that draws from both modern rehabilitation principles and traditional Chinese bone‑setting concepts. From a biomechanical standpoint, the protocol incorporates muscle energy techniques, mobilization with movement, and low‑amplitude joint gapping to progressively correct pelvic dysfunction across the sagittal, coronal, and horizontal planes, while simultaneously addressing segmental lumbar rotation and myofascial compensatory spasm.

From a TCM perspective, the protocol aligns with the classical principle of “bones supporting tendons, and proper bone alignment facilitating tendon flexibility.” It follows a micro‑adjustment, stepwise correction approach and deliberately avoids high‑velocity thrusts. By releasing muscle tension first and then correcting joint alignment, it circumvents the risks of ligamentous injury, joint irritation, and spinal compensatory stress often associated with high‑velocity manipulation—offering enhanced safety and broader clinical applicability. Ultimately, the protocol achieves “global pelvic balance plus segmental lumbar correction” for lumbosacral‑pelvic complex mechanical reconstruction. With its precise assessment, standardized procedure, and strong reproducibility, it is well suited for clinical dissemination and routine application.

Reference：

1. Toyohara R, Ohashi T. A literature review of biomechanical studies on physiological and pathological sacroiliac joints: articular surface structure, joint motion, dysfunction and treatments. Clin Biomech (Bristol, Avon). 2024;114:106233. doi:10.1016/j.clinbiomech.2024.106233.

2. Soomro RR, Karimi H, Gilani SA. Comparative efficacy of quadratus lumborum muscle energy technique with gluteus medius strengthening versus gluteus medius strengthening alone in sacroiliac joint dysfunction: a randomized controlled trial. Diagnostics (Basel). 2024;14(21):2413. doi:10.3390/diagnostics14212413.

3. Mitchell FL Jr, Mitchell PKG. The Muscle Energy Manual: Evaluation and Treatment of the Pelvis and Sacrum. Vol 3. 2nd ed. East Lansing, MI: MET Press; 2001.

4. Gibbons J. Functional Anatomy of the Pelvis and the Sacroiliac Joint: A Practical Guide. Chichester, England: Lotus Publishing; 2016.

5. Yalçınkaya B, Sezgin EA, Saçıntı KG, Özçakar L. Neuromusculoskeletal disorders in pregnancy revisited: insights and clinical implications. Jt Dis Relat Surg. 2025;36(3):741-750. doi:10.52312/jdrs.2025.2242.

6. Lee D, Lee LJ. The Pelvic Girdle. 4th ed. Edinburgh: Elsevier; 2018.

7. Lin ZG, Jiang SC, Cheng YB, et al. Exploring the guiding significance of the "tendon–bone" theory in the Huangdi Neijing for traditional Chinese medicine Tuina. China J Tradit Chin Med Pharm. 2016;31(7):2491-2493.

8. Nordin M, Frankel VH. Basic Biomechanics of the Musculoskeletal System. 5th ed. Philadelphia: Lippincott Williams & Wilkins; 2021.

9. Bogduk N. Clinical and Radiological Anatomy of the Lumbar Spine. 5th ed. Edinburgh: Elsevier/Churchill Livingstone; 2012.

10. Gong GH, Dong BQ, Fu Y, et al. Exploring manual therapy for nonspecific low back pain based on the meridian-tendon theory. Massage Rehabil Med. 2023;14(4):79-81. doi:10.19787/j.issn.1008-1879.2023.04.023.

11. Bordoni B, Sina RE, Varacallo MA. Anatomy, abdomen and pelvis, quadratus lumborum. In: StatPearls [Internet]. Treasure Island, FL: StatPearls Publishing; 2024 Jul 17 [cited 2026 Jun 28]. Available from: [https://www.ncbi.nlm.nih.gov/books/NBK535407/](https://www.ncbi.nlm.nih.gov/books/NBK535407/).

12. Snider EJ, Pamperin K, Pazdernik V, Degenhardt BF. Influence of transverse process landmark localization on palpation accuracy of lumbar spine models. J Am Osteopath Assoc. 2018;118(3):151-158. doi:10.7556/jaoa.2018.034.

13. Henyš P, Ramezani M, Schewitz D, Höch A, Möbius D, Ondruschka B, Hammer N. Sacrospinous and sacrotuberous ligaments influence in pelvis kinematics. J Anat. 2022;241(4):928-937. doi:10.1111/joa.13739.

14. Weis CA, Stuber K, Murnaghan K, Wynd S. Adverse events from spinal manipulations in the pregnant and postpartum periods: a systematic review and update. J Can Chiropr Assoc. 2021;65(1):32-49.

15. Makofsky H. Lumbar spine mobilization/manipulation. In: Makofsky H, editor. Spinal Manual Therapy: An Introduction to Soft Tissue Mobilization, Spinal Manipulation, Therapeutic and Home Exercises. 2nd ed. New York: Routledge; 2024. Chapter 24.

16. Li FJ, Gu JY, Wang CY, et al. Summary of Professor Sun Shuchun's experience in diagnosing and treating lumbar facet joint disorders. J Tradit Chin Orthop Traumatol. 2023;35(6):64-66,69.

17. Pickar JG, Wheeler JD. Response of muscle proprioceptors to spinal manipulative-like loads in the anesthetized cat. J Manipulative Physiol Ther. 2001;24(1):2-11. doi:10.1067/mmt.2001.112017.

18. Jiang HJ, Zhong YM, Huang JF, et al. Clinical effect of stick-point tendon-regulating and bone-setting manipulation based on the theory of "bone alignment and tendon flexibility" in the treatment of acute lumbar sprain. China Med Pharm. 2024;14(5):102-106. doi:10.20116/j.issn2095-0616.2024.05.23.

19. Li CH, Tang LZ, Lin BQ, et al. Effects of reduction manipulation on pelvic and lower extremity biomechanics in sacroiliac joint dysfunction. J Tradit Chin Med Rehabil. 2024;1(4):18-21,25. doi:10.19787/j.issn.2097-3128.2024.04.004.
